# Physicochemical Characterization of Pectic Polysaccharides from Rose Essential Oil Industry By-Products

**DOI:** 10.3390/foods13020270

**Published:** 2024-01-15

**Authors:** Anton Slavov, Vesela Chalova

**Affiliations:** 1 Department of Organic and Inorganic Chemistry, University of Food Technologies, 26 Maritsa Blvd., 4002 Plovdiv, Bulgaria; antons@uni-plovdiv.net; 2 Department of Biochemistry and Molecular Biology, University of Food Technologies, 26 Maritsa Blvd., 4002 Plovdiv, Bulgaria

**Keywords:** rose, rose by-products, by-product valorization, pectin extraction, pectic polysaccharides, rheological studies, food gels

## Abstract

The rose essential oil industry generates large quantities of solid byproducts yearly. These by-products, usually discarded, could yield valuable substances, such as pectic polysaccharides, widely used in the food industry as jelling agents. Seven industrial by-products were investigated as a source of pectic polysaccharides: four samples resulted from the treatment of *Rosa damascena*, two from *Rosa alba*, and one from *Rosa centifolia*. Three by-products were from steam-water distillation, two from CO_2_-supercritical extraction, and two after extraction with hexane and 1,1,1,2-tetrafluoroethane. The by-products were pretreated with 70% ethanol and extracted with 0.1 M HCl. The highest polysaccharide yield was observed for 1,1,1,2-tetrafluoroethane-extracted (RD_F) *Rosa damascena* by-products (13.98 ± 0.14%), followed by hexane (RD_X) and CO_2_-extracted (RD_CO2) *Rosa damascena* (12.68 ± 0.11 and 12.66 ± 0.10%, respectively). The polysaccharides were middle-methoxylated pectins, except RD_F and RD_X, having 26.68 ± 1.14 and 31.39 ± 1.39 mol % degree of methoxylation (low-methoxyl pectins). The polysaccharides had molecular masses in the 2.3–2.6 × 10^4^ Da range. The rheological studies suggested RD_F formed a strong high-sucrose gel, while the others yielded weak gels. RD_F and RD_X formed strong Ca^2+^-mediated gels, comparable with commercial low-methoxylated citrus pectin. This study suggests that rose oil industry by-products could be successfully valorized and yield pectic polysaccharides with gelling properties, comparable with commercial citrus pectins.

## 1. Introduction

Pectin is among the most complex heteropolysaccharides commonly found in higher plant cell walls and middle lamellae [1]. Five major subunits of the pectin macromolecules can be identified: homogalacturonan (HG), xylogalacturonan, apiogalacturonan, rhamnogalacturonan I (RG-I), and rhamnogalacturonan II [2,3]. Except for RG-I, whose backbone consists of repeating units of →4-α-D-galacturonic acid-1 → 2-α-L-rhamnose, the other pectin subunits are built by 1 → 4-linked α-D-galacturonic acid residues. Pectin is widely used in the food industry as a jellifying agent in the preparation of jams and jellies, and as a thickener, emulsifier, texturizer, and stabilizer [4,5]. Pectin is usually added to fruit juices, fruit drink concentrates, desserts, fruit baking preparations, and dairy products [3,6]. It has been used as a fat substitute in spreads, ice cream, and salad dressings [4], and as a carrier of small biologically active molecules for better delivery and accessibility in the digestive tract [7]. Pectin can be obtained from many sources with a variation in the percentage yield, degree of methylesterification, degree of acetylation, and molecular weight. Industrially, pectins are extracted from by-products of the fruit juice industry such as citrus peels and apple pomace by dilute acid extraction [4,8,9]. Other pectin sources include cocoa husks, with about 9% pectin [10], and soy hulls with approximate 26–28% pectin [11]. Sugar beet and sunflower head residue contained 10 to 20% pectin [11]. Despite the clear advantage of citrus peels and apple pomace (abundance, availability, quality of the extracted pectins, etc.) as main pectin raw materials [4], the search for new potential sources continues.

Despite the numerous species of rose grown historically by mankind, only a few have been used for the industrial production of essential oils, namely: *Rosa damascena* Mill. (Damask rose or Bulgarian rose), *Rosa alba* L. (White rose), *Rosa gallica* L. subsp. Eriastyla Kell. var. Austriaca Grantz f. panonica Br (Istanbul rose), *Rosa francofurtana* var. Agatha (Sakar rose), and *Rosa centifolia* [12]. *Rosa alba* plantations previously represented about 40% of all rose plantations, but *Rosa damascena* has become the main plant grown in Bulgaria, due to it having the highest content of rose oil and its superior quality. The differences in the main active and aromatic substances and the relatively low amount of oil in other varieties of roses limit their cultivation, but at least some of them (*Rosa centifolia*, for example) are the main crops grown in Morocco, France, and Egypt. The largest producers in the world are Bulgaria and Turkey, supplying about 80–90% of the rose oil on the market. Other, smaller producers are Iran, Morocco, Mexico, France, Italy, Lebanon, India, Russia, and China. In recent years, countries such as Saudi Arabia, Afghanistan, and Egypt have also contributed to the production of rose oil, and some small plantations have even begun to cultivate roses industrially in Greece and Romania [12,13].

The main purpose of growing the rose species is the production of aroma products, namely: essential oil, water, concrete, absolute, as well as, extracts with organic solvents or liquefied gases [12]. In general, the amount of aroma substances in the plant biomass is low: around 4000 kg of *Rosa damascena* Mill. flowers are needed to produce a kilogram of essential oil and, for other rose species, even more raw material is necessary [13]. This leads to the production of large quantities of solid and liquid by-products. Being considered biodegradable, they are freely discarded near production facilities. However, the liquid part contains higher concentrations of polyphenols, which have a negative influence on the amount of available oxygen in aquatic systems. The solid residues, often used as a fertilizer when bio-certified roses are grown, could introduce unnecessary changes in the soil microbiota [14], and should be preliminary composted [15]. Additionally, the solid by-products are also rich sources of polyphenols, and polysaccharides, which could be further valorized [12]. In previous experiments, Slavov et al. [16] obtained water-soluble pectins from waste rose petals and investigated their immunomodulating properties. Therefore, rose oil industry by-products could be considered a potential source of pectic polysaccharide. Hence, the idea of the present work emerged from the possibility of utilizing solid by-products from the rose oil industry as a source of pectic polysaccharides. A comparison of the physicochemical properties of the polysaccharides isolated from different rose species and by-products resulting from different industrial treatments of the fresh material was made. Their application in food systems as jellifying agents was investigated as well.

## 2. Materials and Methods

By-products from the essential rose-oil industry were collected in the period May–June 2015–2017 and resulted from the processing of flowers of *Rosa damascena* Mill., *Rosa alba,* and *Rosa centifolia* (Table 1). The solid rose by-products, after the distillation/extraction was completed, were removed from the stills, cooled down (if necessary), and inspected to remove unwanted impurities (insects, minerals, weeds, etc.). Then, the residues were pressed to remove the liquid phase (in cases where this resulted from steam-water distillation) and dried in a laboratory dryer at 50 °C, after which the dried by-products were stored at room temperature 18 ± 2 °C until further processing.

Two industrially used citrus pectins for preparation of jams, marmalades, etc., namely: high-methoxyl (HM_CP) pectin type Ceampectin RS-4710 (with a degree of methoxylation (DM) of 75.1 ± 1.1%, and a degree of acetylation (DAc) of 1.2 ± 0.1%) and low-methoxyl (LMA_CP) amidated pectin type GENU^®^ LM-101 AS (with a DM of 34.2 ± 1.1 %, a DAc of 1.1 ± 0.1%, and a degree of amidation (DA) of 14.1 ± 1.2%), were used as controls and were kindly provided by Jam & Jam Ltd. (Plovdiv, Bulgaria).

### 2.1. Preparative Methods

#### 2.1.1. Preparation of the Alcohol Insoluble Part (AIP)

The AIPs were obtained by treatment of the dried rose by-products with 70% ethanol (*v*/*v*). The extraction was carried out in duplicate at 60 °C for 1 h at constant stirring at 150 rpm. In the first extraction, 300 g of rose oil industry by-products were treated with 2000 mL 70% ethanol (*v*/*v*). Then, the mixture was kept for 24 h at room temperature and was filtered through a nylon cloth (250 mesh). A second consecutive extraction was performed with the solid residue under the same conditions, but using 1000 mL of 70% ethanol (*v*/*v*). After the second filtration, the solid residue was dried in a laboratory drier at 50 °C.

#### 2.1.2. Acid Extraction of the AIPs with 0.1 M HCl

The AIP (70.0 g) was treated with 1400 mL 0.1 M HCl (pH 1.2) at 88 °C for 1 h with constant stirring (150 rpm). The plant mass was filtered using a nylon cloth (250 mesh) and the resulting solid residue was extracted with a new portion of 1000 mL 0.1 M HCl at 88 °C for 1 h, stirring at 150 rpm. The mixture was filtered (using a 250 mesh cloth), both filtrates were combined and the volume was reduced under vacuum to 1/2 of its initial value. The concentrated filtrate was precipitated with 96% ethanol (*v*/*v*) (1:3 volume:volume) overnight at 4 °C (final ethanol concentration 75%), and the precipitate was centrifuged (5000 rpm, 5 °C, 30 min). The precipitate was dissolved in 80 mL deionized water and dialyzed (Spectra/Por 1, Breda, The Netherlands; which retains substances with molecular weight higher than 6–8 kDa) for 72 h against deionized water. The content of the dialysis membrane was lyophilized and denoted as acid-extractable polysaccharides (AEP).

### 2.2. Analytical Methods

The degree of esterification (DE) and the polyuronide content (PUc) of the rose oil industry by-products and the AIPs were determined titrimetrically as described by Slavov et al. [17]. The amount of ash was determined after heating the samples (1 g of each) at 605 °C until they reached a constant weight using a laboratory muffle furnace (MLW 212.11, MLW, Germany). The protein content in the by-products and the AIPs (1 g of each) was assessed by an automatic Kjeldahl nitrogen analyzer MultiKjel-365 equipped with an automated Metrohm ECO titrating unit (Buchi Labortechnik, Flawil, Switzerland) using 6.25 as a multiplication factor.

The amount of proteins co-extracted with the polysaccharides (100 μL of 1 mg/mL polysaccharide solution in deionized water) was determined spectrophotometrically with an AMRESCO E535-KIT (AMRESCO, Solon, OH, USA) employing as standard bovine gamma-globulin. The anhydrouronic acid content (AUAC) of the polysaccharides (50 μL of a 1 mg/mL polysaccharide solution in deionized water) was investigated by the *m*-hydroxydiphenyl method using D-galacturonic acid as a standard [18]. The total amount of neutral sugars of the polysaccharides (100 μL of a 1 mg/mL polysaccharide solution in deionized water) was investigated by the phenol-sulfuric acid method [19].

The DM and the DAc were determined by quantification of the methanol and acetic acid, respectively, released by alkaline de-esterification of the polysaccharides. A 5 mg polysaccharide sample was suspended in solution containing 25 μL of iso-propanol as an internal standard and 0.5 mL of 1 M NaOH for 1 h at 4 °C in the presence of 5 mM of CuSO_4_ (CuSO_4_, Merck, Darmstadt, Germany). An ELITE La Chrome (Hitachi, Tokyo, Japan) HPLC with a VWR Hitachi Chromaster 5450 refractive index detector (RID) equipped with a C18 Superspher column (Merck, Germany) using 4 mM H_2_SO_4_ as the mobile phase at a 0.7 mL/min flow rate (25 °C temperature column and 35 °C temperature of the RID) was used for the determination of the amount of methanol and acetic acid. Isopropanol was used as an internal standard, and DM and DAc were calculated as the molar ratio of methanol and acetic acid, respectively, to 100 galacturonic acid units.

The monosaccharide composition of the isolated pectic polysaccharides was determined as follows: 20 mg of polysaccharide was hydrolyzed with 25 mL of 2 M trifluoroacetic acid (Sigma-Aldrich Chemie Gmbh, Steinheim am Albuch, Germany) for 3 h at 120 °C. The mixture was evaporated to dryness under a vacuum, washed with 10 mL of deionized water, and again evaporated to dryness. The washing was performed three times to remove the residual trifluoroacetic acid. After the last evaporation step, the hydrolysates were dissolved in 1.5 mL of deionized water. The neutral sugars and uronic (galacturonic and glucuronic) acids were determined on an ELITE La Chrome (Hitachi, Japan) HPLC with a VWR Hitachi Chromaster 5450 RID using an Aminex HPX-85H column. The mobile phase used for the elution of the samples and standards was 5 mM of H_2_SO_4_ (Sigma-Aldrich Chemie Gmbh, Germany) at a 0.5 mL/min rate. The column temperature was set at 50 °C, and the detector temperature at 35 °C. The determination of xylose and mannose was performed in a separate run using the same chromatographic system equipped with a Sugar SP0810 (Shodex^®^ Showa DENKO, Tokyo, Japan) column. The elution was performed with ultrapure water at an elution rate of 1.0 mL/min. The column temperature was set to 85 °C and the RID temperature was 35 °C.

The polysaccharides’ molecular mass was determined using an ELITE La Chrome (Hitachi, Japan) with a VWR Hitachi Chromaster 5450 RID (Hitachi, Japan). The separation was performed on an OHpak SB-806 M (Shodex^®^ Showa DENKO, Japan) column. The elution of the samples (dissolved at 4 mg/mL in the mobile phase) and standards (dissolved in the mobile phase at 4 mg/mL) was performed in isocratic mode using NaNO_3_ (0.1 mol/L) at a 0.8 mL/min rate. The column temperature was set to 30 °C and the detector temperature to 35 °C. The column was equilibrated with pullulans (Shodex^®^ Showa DENKO, Japan) with the following molecular masses: 6.2, 10.0, 21.7, 48.8, 113.0, 200.0, 366.0, and 805.0 kDa, used to prepare the standard curve for determination of the polysaccharides’ molecular masses.

### 2.3. Rheological Studies

Rheological investigations of the polysaccharides’ gelling behavior were performed at 25 °C with a controlled stress rheometer (AR-G2, TA instruments, New Castle, DE, USA) equipped with a built-in Peltier temperature control unit. A cone and plane geometry of 60 mm diameter, 1° angle (with the gap between cone and plane during measuring 150 µm) was used. Two types of systems, representing the main type of gels with pectins being produced industrially, were investigated: high-sucrose (60% sucrose gels (*w*/*w*)) and Ca^2+^ (6 mmol/L)-induced gels.

(1) Preparation of the high-sucrose gel: The polysaccharide solution (2.5%) was prepared by dissolving 0.25 g of polysaccharide in a 10 mL 50 mmol/L citrate buffer with pH 3.5 by stirring overnight. The solution was heated to 90 °C and 15 g of sucrose was added in portions and dissolved by stirring (the final concentrations of polysaccharide and sucrose were 1.25% and 60% (*w*/*w*), respectively).
(2) Ca^2+^-induced gel preparation: The polysaccharide solution (2.5%) was prepared by dissolving 0.25 g of polysaccharide in a 10 mL 50 mmol/L citrate buffer with pH 3.5 by stirring overnight. The solution was heated to 90 °C and the 10 mL 12 mmol/L CaCl_2_ solution (heated to 90 °C) in 50 mmol/L citrate buffer (pH 3.5) was slowly added to the polysaccharide solution (final concentrations of polysaccharide and Ca^2+^: 1.25% (*w*/*w*) and 6 mmol/L, respectively).
(3) Rheological studies were then performed as follows: 2 mL of 25% polysaccharide solution (preheated at 90 °C) was pipetted and evenly spread on the rheometer plate (heated at 90 °C). The cone was lowered to the desired position and the sample was immediately covered with oil to prevent the evaporation of water. The temperature was decreased to 25 °C with a 7 °C/min rate and the measurements were started. The mechanical spectra of the gels were recorded by measuring the storage (G′) and loss moduli (G″) at 1 rad/s frequency and 1% strain amplitude. After the time sweep test finished it was followed by a frequency sweep test at the same deformation rate.

### 2.4. Statistical Analysis

The experiments were performed in triplicate and values were expressed as mean ± standard deviation (SD). Statistical analysis was carried out by one-way ANOVA (Tukey’s post hoc test; *p* < 0.05) using Microsoft Excel 2013 with an added XL Toolbox NG application.

## 3. Results and Discussion

The by-products were subjected to preliminary analyses for the determination of proteins (by the Kjeldahl method), ash, and polyuronide content, and their respective degree of esterification (Table 2).

The main type of industrial processing of essential oil rose flowers is performed in large stills by steam-water distillation. The still is loaded with the flowers from bags and water is added (roughly the ratio is fresh roses: water = 1:5). Then, the mixture is heated by steam and the distillation takes place for 2 to 2.5 h. After finishing the process, the mass from the still is pumped out and passes through a screw conveyor where most of the liquid is removed [20]. For the other types of treatment of roses—extraction by hexane, 1,1,1,2-tetrafluoroethane subcritical extraction, and CO_2_ supercritical extraction—the temperature is relatively low (below 60 °C). This suggested that the by-products from the steam-water distilled roses could have undergone more substantial degradation during the heat treatment. Furthermore, due to the water present in the still, although the ratio is not optimal for pectin extraction (usually the ratio of raw material to water is around 1:20 [4]) partial solubilization of pectic polysaccharides could take place. Examining the results for PUc of the by-products from *Rosa damascena* Mill. (Table 2; by-products 1–4) it could be seen that the lowest values were observed for steam-distilled residue. The highest PUc value of RD_CO2 suggested that the supercritical CO_2_ extraction which took place at lower temperatures preserved, to a large extent, the polyuronides in the cell walls. Additionally, the treatment of plant material with supercritical liquefied gases is known to disrupt the cell walls and, hence, facilitate further extraction of substances from the cells [17]. The same conclusion could be drawn comparing the PUc results for RA_CO2 and RA_SD (6.33 ± 0.19% and 5.41 ± 0.49%, respectively). A similar observation was made by Slavov et al. [17] investigating two marigold wastes: one resulted from steam-water distillation (HD) and the second resulted from subcritical extraction with 1,1,1,2-tetrafluoroethane (F). The polyuronic acid content in the F residue was twice as high as HD wastes, suggesting substantial solubilization and extraction of the protopectic polysaccharides present in the cell walls.

Treatment of the by-products with 70% ethanol resulted in the preparation of AIP. This treatment, on the one hand, aimed to remove low-molecular accompanying substances (pigments, polyphenols, sugars, aroma compounds, glycosides, etc.) that remained after industrial processing. These compounds would interfere with the further extraction of polysaccharides in an acidic medium. On the other hand, the extracts, obtained as a result of the ethanol pretreatment, contain polyphenolic substances, which could be valorized as potential antioxidant dietary supplements [12]. The characteristics of the AIPs, obtained by treatment of the by-products with 70% ethanol, are presented in Table 3.

The same conclusion, which was made for the by-products, could be drawn by examining the values for the PUc of the AIPs: the AIPs which resulted from steam-water distillation by-products had lower values than the AIPs obtained from extraction (supercritical CO_2_ extraction, hexane extraction, and 1,1,1,2-tetrafluoroethane subcritical extraction) procedures. The highest PUc was observed for the samples RA_CO2, RD_X, and RD_CO2 (6.77 ± 0.14%, 6.36 ± 0.27%, and 6.30 ± 0.48%, respectively). The ash content was similar for all the investigated AIPs and the protein content was in the 12.67 ± 0.40%–18.34 ± 0.25% range.

The alcohol-insoluble parts of the by-products obtained as a result of treatment with a 70% ethanol solution were subjected to acid extraction and acid-soluble polysaccharides (AEP) were obtained. The polysaccharides were subjected to analyses for determination of their composition and physicochemical parameters. The results of the AEPs’ yields, as well as data on the anhydrouronic acids, neutral sugars, and protein content are summarized in Table 4. The results for the degree of methoxylation and amidation, molecular mass, and the polysaccharides’ heterogeneity, determined by gel chromatography, are also presented in Table 4.

From the data presented, it can be concluded that the polysaccharides from *Rosa damascena* by-products obtained after extraction with hexane, 1,1,1,2-tetrafluoroethane, and carbon dioxide had the highest total yield, followed by the polysaccharides from *Rosa alba* by-products obtained after extraction with carbon dioxide. Polysaccharides from *Rosa damascena* by-products (RD_CO2) extracted with carbon dioxide are distinguished by the highest content of anhydrouronic acids (747.12 ± 14.23 µg/mg polysaccharide), neutral sugars (881.13 ± 18.29 µg/mg polysaccharide), and protein (121.65 ± 6.33 µg/mg polysaccharide). All the AEPs were middle methoxylated pectic (around 50 mol % DM) polysaccharides, except RD_F and RD_X, which had DM (26.68 ± 1.14 and 31.39 ± 1.39 mol %), characteristic for low methoxylated pectic polysaccharides. The results suggested that the obtained pectic polysaccharides had similar molecular masses: in the 2.3–2.6 × 10^4^ Da range. The polysaccharides obtained from *Rosa alba* by-products were heterogeneous and had an around 10% higher molecular weight polysaccharide fraction and the other 90% had molecular mass comparable to the other AEPs.

Furthermore, the obtained pectic polysaccharides were hydrolyzed with trifluoroacetic acid and the resulting hydrolysates were subjected to chromatographic separation to determine the monosaccharide composition of AEPs (Table 5).

The data for the monosaccharide composition and the high GalA content (for all the samples above 600 µg/mg polysaccharide), combined with those for the content of uronic acids (determined spectrophotometrically by the formation of colored products with *m*-hydroxydiphenyl), suggested that the isolated polysaccharides were typical pectic polysaccharides. Galactose, from the neutral monosaccharides, stands out with the highest content in all the AEPs, followed by arabinose, which indicated the presence of side chains made up mainly of galactose residues, and also the presence of arabinan and arabinogalactan side chains [21]. Additionally, the low pH and elevated temperatures favor trimming the side chains during extraction and increasing the final amount of GalA in the extracted polysaccharides [8]. The ratio GalA/Rha, which in general provides an overview of the HG/RG-I ratio in the AEPs [8], suggested the presence of RG-I around 10–14% and the other part consisted of HG and RG-II subunits. Similar results for RG-I content in pectic polysaccharides isolated from grape and soybean pomace, 10 and 15%, respectively, were reported by Voragen et al. [3]. The low amounts of xylose suggested that xylogalacturonans were absent or could be found in very low amounts. The sum of Ara + Gal amounts divided on Rha could provide insights on the RG-I branching (amount and type of neutral sugar side chains) [8]. The calculated (Ara + Gal)/Rha for the rose AEPs were in the 4.00–5.36 range, which is characteristic of pectins extracted by acids (nitric, oxalic, etc., using harsh and medium conditions) [8,22]. Applying less harsh extraction conditions (extractants: water; chelating reagents, such as oxalates, cyclohexane-1,2-diamine-tetraacetic acid, or alkaline carbonates; and lower temperatures) leads to higher (Ara + Gal)/Rha ratios [22], which corresponded to pectins with higher branching of RG-I (higher amounts of neutral sugar side chains). The gelation ability of pectins is mostly related to the HG subunit and, hence, rheological studies with AEPs were performed in the subsequent experiments.

### Rheological Investigations

Pectin is widely used in the food industry as a gelling agent [4]. The gelation of pectin polysaccharides depends on several important parameters, such as the degree of methoxylation (including the distribution pattern of the free carboxyl groups), degree of acetylation, molecular weight, and conditions of the food system (pH, presence of co-solutes, temperature, etc.), and can be carried out through two main mechanisms [23,24,25]. Highly methoxylated pectins tend to form gels at a lower pH (2.0–3.7) in the presence of hydrophilic low-molecular weight co-solvents (an approach primarily used in the food industry for the preparation of high-sucrose gels), while low-methoxylated pectins or low-methoxylated amidated pectins can gel at higher pH levels, but need the presence of divalent cations, such as calcium, to form gels [3].

**Figure 1:**
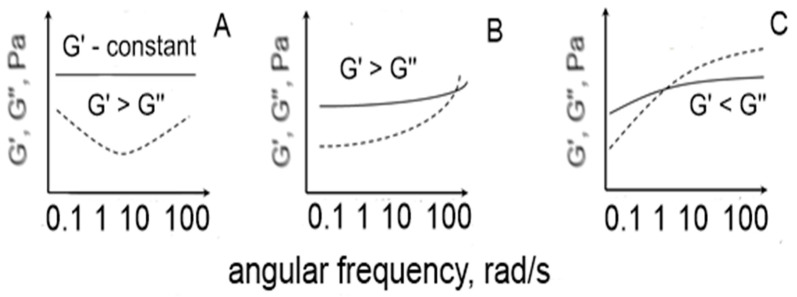
Gelling behavior of pectic polysaccharides depending on how the G′ (solid line) and G″ (dotted line) values change with increasing angular frequency. (**A**) strong gel; (**B**) weak gel; (**C**) macromolecular solution.

The techno-functional properties of pectic food-type systems were studied by measuring the storage (G′) and loss modulus (G″) changes with time (the evolution of gels) and the increases in the angular frequency (the mechanical spectra of gels). Depending on the changes in the values of G′ and G″, and the increases in the angular velocity during the frequency sweep test, three types of pectic food systems could be distinguished (Figure 1). When G′ > G″ (and G′ had a constant character at the higher values of the angular velocity) the resulting system could be classified as strong gel; for G′ > G″ (and G″ having higher values than G′ at the higher values of angular velocity) the resulting system is a weak gel; and in the third case when G′ < G″ the resulting system is a macromolecular viscous (non-Newtonian) type of solution [26].

For this reason, to investigate the viscoelastic properties of the isolated rose AEPs, in the following experiments rheological studies were performed using 1.25% (final concentration) pectic polysaccharide food-type gel systems at pH 3.5.

**Figure 2:**
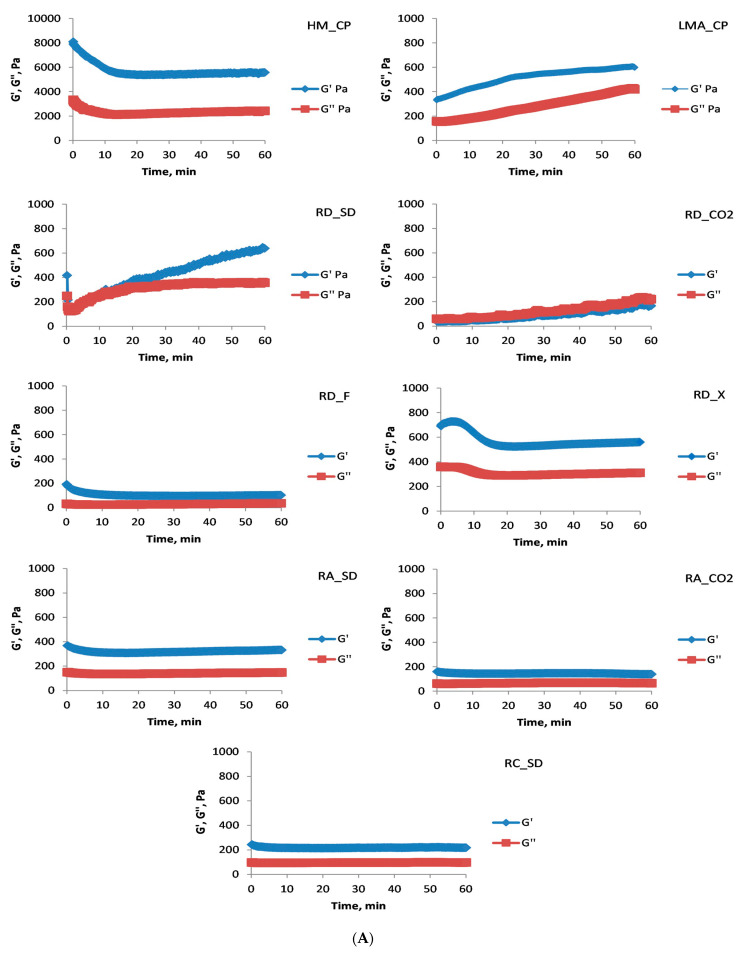

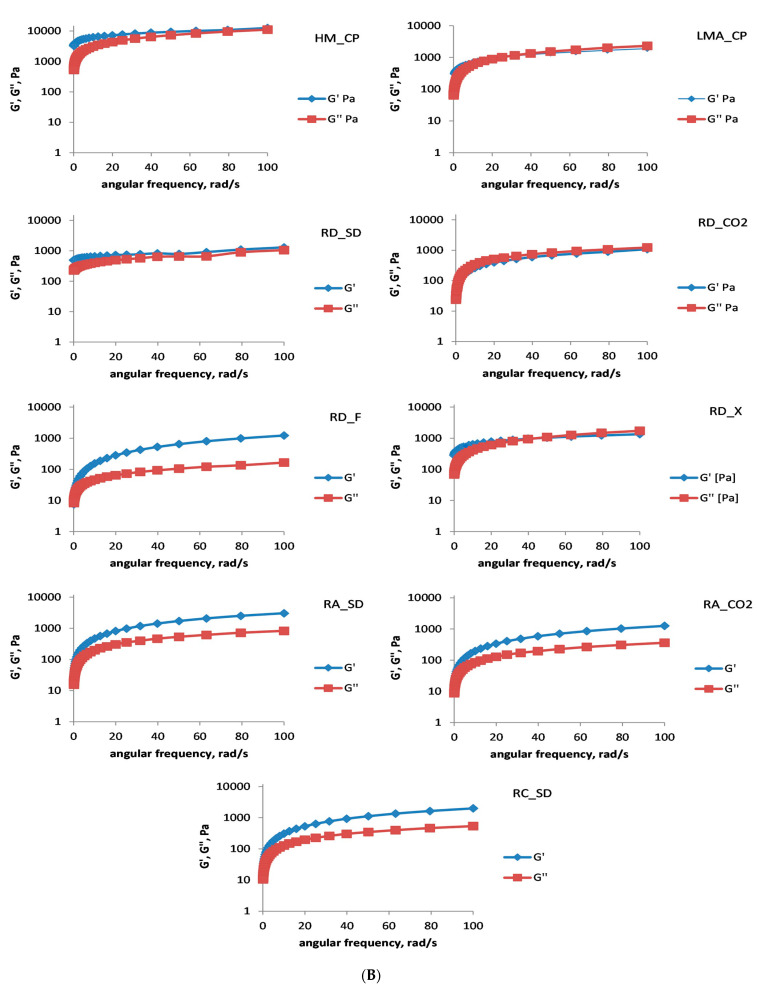
Rheological studies of high-sucrose gels (polysaccharide concentration: 1.25% (*w*/*w*); concentration of sucrose 60% (*w*/*w*); medium: 50 mmol/L citrate buffer, pH 3.5). (**A**) Time sweep test, (**B**) mechanical spectra of gels. Thin lines—G′, thick lines—G″.

Initially, high-sucrose food systems (60% sucrose) were investigated, and an oscillatory test (time sweep test) was conducted at a constant strain amplitude of 1% (Figure 2A). After lowering the temperature, pectic polysaccharides began to form three-dimensional structures, which, depending on the polysaccharide, resulted in gels or viscous solutions. All of the investigated pectin polysaccharides obtained from the by-products of the rose oil industry showed that under the given conditions they could form high-sucrose gels, and at the end of the measurement (60 min) the evolution of the gel system was almost complete (G′ values were constant) (Figure 2A).

The gel obtained from the commercial HM citrus pectin (HM_CP) was distinguished by the highest values of G′. It should be noticed, however, that the HM_CP had the highest GalA content (812.05 ± 2.14 µg/mg polysaccharide), the highest DM (75.1 ± 1.1%), the lowest DA (1.2 ± 0.1%), and the average molecular weight was an order of magnitude higher (5.9 × 10^5^ Da) than all the rose by-product polysaccharides. After the time sweep test was completed, an analysis of the mechanical strength of the resulting gel systems was conducted (Figure 2B) by varying the cone angular velocity from 0.1 to 100 rad/s. The established dependencies on the changes in the G′ and G″ values at low and high angular velocities are indicative of the resulting product, either a polysaccharide-derived gel (strong or weak) or a macromolecular viscous solution. For all the polysaccharides studied, the formation of a gel was observed. The systems, except for RD_CO2, RD_SD, and RD_X, were strong gels and were comparable with the gelation (at the given conditions) of the LMA_CP. For all the isolated polysaccharides, G′ is one order of magnitude lower than that of commercial HM_CP. It has to be mentioned, however, that the pH value (50 mmol/L citrate buffer, pH 3.5) was slightly higher than the optimum conditions for the gelation of HM pectins [26,27,28]. The strength of the gels RD_F, RC_SD, RA_SD, and RA_CO2, on the other hand, was comparable with gel systems obtained under the same conditions with pectins isolated from marigold waste [17], as well as pectin isolated from chicory roots using “green” (enzymatic) treatment methods [26]. The results suggested that *Rosa amascene* pectic polysaccharides showed the worst gelling properties, except for RD_F, where tentatively the extraction with 1,1,1,2-tetrafluoroethane (mild conditions) could be the reason for the strong high-sucrose RD_F gels. It is difficult, however, to unequivocally draw conclusions about the influence of the type of initial raw material or type of industrial treatment on the gelation pattern of the isolated polysaccharides. Having in mind that the molecular weights of the rose pectic polysaccharides were in the same range (2.3–2.6 × 10^4^ Da), it seemed that the DM and Dac could be the most influential factors for gelation in the presence of 60% sucrose at pH 3.5. It should also be noticed that RD_F, RC_SD, RA_SD, and RA_CO2 were able to produce strong high-sucrose gels at 1.25% polysaccharide concentration, which is twice lower than the gels obtained with ponkan (*Citrus reticulata* blanco cv. *Ponkan*), pectin (optimal pH 2.5, polysaccharide concentration 2.5%) [27], or gels obtained with pectin enriched fractions at 2 and 5% polysaccharide concentrations [29].

**Figure 3:**
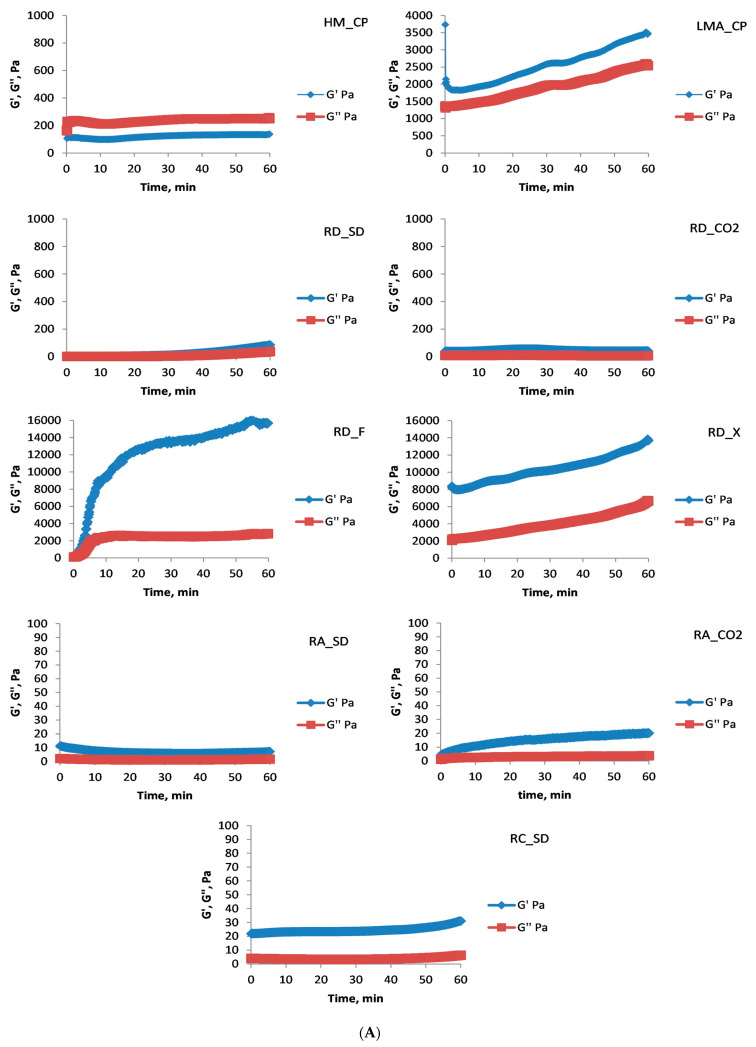

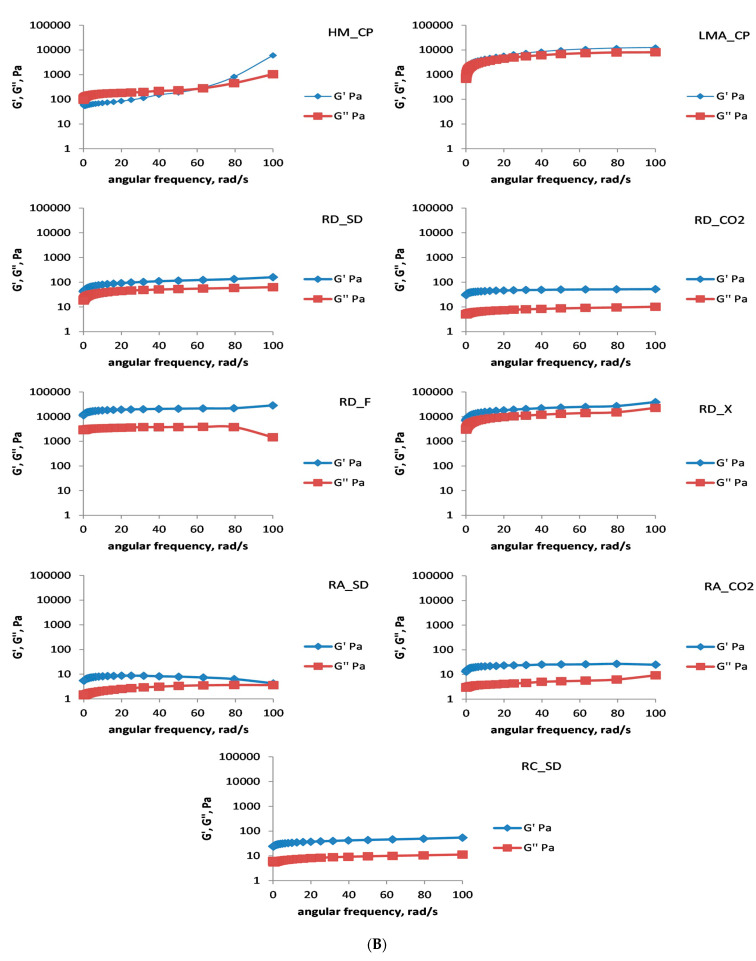
Rheological studies of Ca^2+^-mediated gels (polysaccharide concentration: 1.25% (*w*/*w*); concentration of Ca^2+^: 6 mmol/L; medium: 50 mmol/L citrate buffer, pH 3.5). (**A**) Time sweep test, (**B**) mechanical spectra of the gels. Thin lines—G′, thick lines—G″.

In the subsequent experiments, the gelation of rose AEPs in the presence of Ca^2+^ (6 mmol/L final concentration) was investigated. The time sweep test (Figure 3A) unequivocally demonstrated that the DM of the polysaccharides was the most important factor governing their gelation behavior in the presence of divalent cations. For all the polysaccharides, except for LMA_CP, RD_F, and RD_X, after 60 min the evolution of the gel had been mostly completed. The commercial low-methoxyl amidated citrus pectin and the two abovementioned rose pectins, having less esterified carboxyl groups, were able to exchange Ca^2+^ and to continuously evolve their three-dimensional structure with time. One of the advantages here for the LMA_CP was slower gel evolution, probably due to the presence of amidated carboxyl groups which lowered the gelation speed, whereas the RD_F (especially) and RD_X quickly formed gels.

The following frequency sweep test (Figure 3B) clearly demonstrated the formation of strong gels by RD_F and RD_X (stronger even than the commercial LMA_CP gel). Not surprisingly, due to the unfavorable conditions for gelation of high-methoxyl pectins (absence of hydrophilic co-solutes), the HM_CP formed a week gel. The RA_SD AEP did not gel in the presence of calcium. All the other AEPs produced strong gels but some of them (RD_SD, RD_CO2, RA_CO2, and RC_SD) were prone to syneresis and water was lost from the gel matrix with time. Jong et al. [30] investigated the rheological properties of durian rind pectin and found that it could gel at 50 mg Ca^2+^/g pectin and 30% sucrose (*w*/*v*), while the optimal conditions were pH 3 and 3.0% polysaccharides. Lin et al. [31], studying the visco-elastic behavior of pectin isolated from passion fruit peels extracted by high-speed shearing, found that at 100–200 CaCl_2_ mg/100 mL of polysaccharide solution the liquid-type system was transformed in sol. The G′ dominated over G″ but at higher Ca^2+^ concentrations (300 mg/100 mL of polysaccharide solution) the observed structure a macromolecular solution. It has to be noted, however, that the molecular weight of the isolated pectin was quite low (12.77 ± 1.36 kDa) compared to classically extracted pectins from the same raw material and commercial citrus pectin (24.09 ± 0.69 kDa) used as a control. Moreover, the authors did not present any information about DM and DAc for the pectic polysaccharides used in the investigation. Comparing the results of the present study with results for gelation behavior of pectic polysaccharides isolated from industrial lavender by-products [32] it could be concluded that the rose AEPs showed better gelation properties in the presence of Ca^2+^ than lavender pectic polysaccharides. The rheological properties of lavender pectins render them unsuitable for the preparation of high-sucrose and Ca^2+^-mediated gels, while rose pectic polysaccharides could be used for the preparation of food-type gel systems with properties comparable to commercial citrus pectins. It would be interesting to investigate further the rheological behavior of the rose AEPs in mixed-type systems: in the presence both of Ca^2+^ and sucrose.

## 4. Conclusions

The results of the present comparative study suggest that by-products from the rose oil industry could be successfully valorized and utilized as a source of pectic-type polysaccharides. The by-products resulting from industrial steam-distillation of fresh rose petals had lower amounts of polyuronides due to the presence of water during the treatment of the material, which could result in partial solubilization of the pectic polysaccharides. The yield of AEPs, however, was similar for all seven AIPs, slightly higher and statistically different for RD_F. The monosaccharide composition revealed that the GalA was the predominant sugar; the RD_CO2 being the pectic polysaccharide with the highest amount of GalA (715.58 ± 8.58 µg/mg polysaccharide). The latter AEP was also characterized by a higher content of uronic acids (747.12 ± 14.23 µg/mg polysaccharide), neutral sugars (881.13 ± 18.29 µg/mg polysaccharide), and protein (121.65 ± 6.33 µg/mg polysaccharide). All of the polysaccharides, except RD_F and RD_X (with DM 26.68 ± 1.14 and 31.39 ± 1.39 mol %, respectively), were middle-esterified pectins. The DAc was low: in the 2.61 ± 0.16–3.37 ± 0.21 mol % acetyl group range, and the molecular weights were in the 2.3–2.6 × 10^4^ Da range.

The rheological studies of the two types of food gels, high-sucrose and Ca^2+^ -mediated gels, unequivocally suggested that the AEPs extracted from by-products of the rose oil industry possess satisfactory gelling properties comparable to commercial low-methoxyl amidated citrus pectin and some of them can be used as jellifying agents and viscosity modifiers for various food systems. The formation of high sucrose gels was observed in all seven AEPs studied. The systems, except for RD_CO2, RD_SD, and RD_X, were strong gels and were comparable with the gelation (at the given conditions) of the control LMA_CP. The RD_F and RD_X AEPs, having the lowest DM (26.68 ± 1.14 and 31.39 ± 1.39 mol % methoxylated carboxyl groups, respectively), showed excellent jellifying abilities in the presence of Ca^2+^ and could be used for formulations in low-calorie preserves.

## Figures and Tables

**Table 1:** By-products of the rose oil industry used in the present study.

By-Product	Species	Type of Industrial Treatment	Harvest	Distillery/Location
1. RD_SD	*Rosa damascena* Mill.	Steam-water distillation	2017	ECOMAAT Ltd., Mirkovo, Sofia, Bulgaria
2. RD_CO2	*Rosa damascena* Mill.	CO_2_ supercritical extraction	2017	ECOMAAT Ltd., Mirkovo, Sofia, Bulgaria
3. RD_F	*Rosa damascena* Mill.	1,1,1,2-tetrafluoroethane subcritical extraction	2017	University of Food Technologies, Department of Industrial Heat Engineering, Plovdiv, Bulgaria (Nenov, 2006)
4. RD_X	*Rosa damascena* Mill.	Hexane extraction	2017	Galen-N Ltd., Zelenikovo, Brezovo, Bulgaria
5. RA_SD	*Rosa alba*	Steam-water distillation	2015	Enio Bonchev Production Ltd., Tarnichene, Kazanlak, Bulgaria
6. RA_CO2	*Rosa alba*	CO_2_ supercritical extraction	2016	ECOMAAT Ltd., Mirkovo, Sofia, Bulgaria
7. RC_SD	*Rosa centifolia*	Steam-water distillation	2015	Enio Bonchev Production Ltd., Tarnichene, Kazanlak, Bulgaria

**Table 2:** General characteristics of the rose oil industrial by-products.

By-Product	Protein, %	Ash, %	DE, %	PUc, %
1. RD_SD	14.21 ± 0.98 ^a^	2.65 ± 0.03 ^d^	58.11 ± 1.91 ^a^	6.79 ± 0.31 ^c^
2. RD_CO2	11.20 ± 0.69 ^b^	4.37 ± 0.08 ^a^	44.12 ± 1.39 ^b^	9.27 ± 0.42 ^a^
3. RD_F	13.81 ± 0.87 ^a^	3.70 ± 0.09 ^b^	45.05 ± 0.85 ^b^	8.24 ± 0.14 ^b^
4. RD_X	10.66 ± 0.86 ^b^	2.75 ± 0.25 ^d^	30.85 ± 0.81 ^d^	8.32 ± 0.33 ^b^
5. RA_SD	14.61 ± 0.69 ^a^	2.71 ± 0.06 ^d^	62.83 ± 2.77 ^a^	5.41 ± 0.49 ^d^
6. RA_CO2	11.30 ± 0.93 ^b^	2.86 ± 0.15 ^d^	60.34 ± 3.05 ^a^	6.33 ± 0.19 ^c^
7. RC_SD	6.81 ± 0.58 ^c^	3.25 ± 0.08 ^c^	36.38 ± 1.83 ^c^	5.65 ± 0.76 ^d^

The values are presented as mean ± standard deviation; ^a,b,c,d^ Different letters in a column show significantly different values (one-way ANOVA Tukey HSD test: *p* < 0.05).

**Table 3:** General characteristics of the AIPs obtained by treatment of the by-products with 70% ethanol.

AIP	Protein, %	Ash, %	DE, %	PUc, %
1. RD_SD	13.35 ± 0.25 ^d,e^	2.42 ± 0.20 ^d^	84.71 ± 0.81 ^a^	4.57 ± 0.14 ^c^
2. RD_CO2	13.60 ± 0.12 ^d^	3.68 ± 0.04 ^b^	63.86 ± 1.35 ^d^	6.30 ± 0.48 ^a^
3. RD_F	15.90 ± 0.31 ^b^	4.26 ± 0.04 ^a^	68.22 ± 1.17 ^c^	5.05 ± 0.23 ^b^
4. RD_X	12.67 ± 0.40 ^e^	3.53 ± 0.24 ^b^	56.17 ± 1.58 ^e^	6.36 ± 0.27 ^a^
5. RA_SD	15.97 ± 0.12 ^b^	2.61 ± 0.06 ^d^	76.59 ± 0.79 ^b^	4.33 ± 0.11 ^d^
6. RA_CO2	18.34 ± 0.25 ^a^	3.12 ± 0.05 ^c^	83.30 ± 1.12 ^a^	6.77 ± 0.14 ^a^
7. RC_SD	14.30 ± 0.23 ^c^	3.48 ± 0.04 ^b^	63.76 ± 0.74 ^d^	3.88 ± 0.25 ^e^

The values are presented as mean ± standard deviation; ^a,b,c,d,e^ Different letters in a column show significantly different values (one-way ANOVA Tukey HSD test: *p* < 0.05).

**Table 4:** Characteristics of the acid-extractable polysaccharides (AEPs).

AEP	Yield, %	Anhydrouronic Acids *, µg/mg	Neutral Sugars, µg/mg	Proteins, µg/mg	DM, mol %	DAc, mol %	Mw, Da	Polysaccharides’ Heterogeneity, %
1. RD_SD	12.45 ± 0.12 ^b^	699.05 ± 10.24 ^b^	745.76 ± 16.17 ^d^	61.15 ± 5.84 ^b^	56.15 ± 1.25 ^a^	2.61 ± 0.16 ^c^	2.31 × 10^4^	100
2. RD_CO2	12.66 ± 0.10 ^b^	747.12 ± 14.23 ^a^	881.13 ± 18.29 ^a^	121.65 ± 6.33 ^a^	59.27 ± 1.67 ^a^	3.14 ± 0.11 ^a^	2.36 × 10^4^	100
3. RD_F	13.98 ± 0.14 ^a^	677.51 ± 12.58 ^b^	818.19 ± 12.38 ^c^	115.75 ± 8.21 ^a^	26.68 ± 1.14 ^d^	3.06 ± 0.31 ^a,b^	2.41 × 10^4^	100
4. RD_X	12.68 ± 0.11 ^b^	689.56 ± 10.11 ^b^	857.63 ± 13.54 ^b^	110.85 ± 7.26 ^a^	31.39 ± 1.39 ^c^	3.37 ± 0.21 ^a^	2.29 × 10^4^	100
5. RA_SD	12.03 ± 0.09 ^c^	589.58 ± 13.36 ^c^	754.92 ± 14.69 ^d^	50.55 ± 5.35 ^b^	49.09 ± 1.57 ^b^	2.23 ± 0.15 ^d^	2.59 × 10^4^ 2.32 × 10^4^	7.87 92.13
6. RA_CO2	10.54 ± 0.12 ^d^	697.69 ± 12.14 ^b^	744.41 ± 15.11 ^d^	117.80 ± 6.59 ^a^	48.86 ± 1.14 ^b^	2.72 ± 0.12 ^c^	2.58 × 10^4^ 2.35 × 10^4^	8.49 91.51
7. RC_SD	12.63 ± 0.13 ^b^	694.37 ± 11.54 ^b^	841.86 ± 16.27 ^b^	113.90 ± 8.36 ^a^	51.46 ± 1.28 ^b^	2.88 ± 0.15 ^b,c^	2.36 × 10^4^	100

* The quantity of anhydrouronic acids was determined spectrophotometrically by the *m*-hydroxydiphenil method; DM—degree of methoxylation; DAc—degree of acetylation; the values are presented as mean ± standard deviation; ^a,b,c,d^ Different letters in a column show significantly different values (one-way ANOVA Tukey HSD test: *p* < 0.05).

**Table 5:** Monosaccharide composition of the AEP µg/mg polysaccharide.

AEP	GalA	GlcA	Rha	Gal	Ara	Glc	Xyl	Man	Fuc	GalA/Rha	(Ara + Gal)/Rha
1. RD_SD	658.36 ± 9.25 ^c^	25.13 ± 2.14 ^b,c^	54.78 ± 3.47 ^a^	191.24 ± 8.37 ^b^	51.47 ± 6.31 ^b,c^	10.24 ± 2.11 ^a,b^	1.24 ± 0.47 ^a,b^	0.48 ± 0.10 ^b^	1.17 ± 0.10 ^a^	12.02	4.43
2. RD_CO2	715.58 ± 8.58 ^a^	19.37 ± 1.23 ^d^	55.47 ± 2.68 ^a^	150.47 ± 9.34 ^c^	71.48 ± 8.26 ^a^	11.02 ± 1.86 ^a,b^	0.99 ± 0.12 ^b^	nd	nd	12.90	4.00
3. RD_F	647.29 ± 8.64 ^c^	27.84 ± 1.86 ^a,b^	50.12 ± 2.87 ^a,b^	180.98 ± 7.29 ^b^	65.24 ± 7.47 ^a,b^	9.84 ± 1.74 ^a,b^	1.01 ± 0.20 ^a,b^	1.05 ± 0.11 ^a^	nd	12.91	4.91
4. RD_X	671.63 ± 9.69 ^b^	31.68 ± 2.47 ^a^	51.39 ± 3.05 ^a,b^	178.36 ± 7.21 ^b^	60.84 ± 6.95 ^a,b^	8.37 ± 1.69 ^b^	1.14 ± 0.24 ^a,b^	1.14 ± 0.09 ^a^	nd	13.07	4.65
5. RA_SD	598.57 ± 8.24 ^d^	24.10 ± 1.96 ^b,c^	48.29 ± 2.47 ^b^	210.43 ± 8.05 ^a^	48.79 ± 5.86 ^b,c^	11.27 ± 2.01 ^a,b^	1.57 ± 0.27 ^a^	nd	1.07 ± 0.09 ^a^	10.32	5.36
6. RA_CO2	675.84 ± 8.47 ^b^	19.84 ± 2.04 ^d^	47.38 ± 2.39 ^b^	187.85 ± 8.11 ^b^	40.28 ± 6.13 ^c^	12.24 ± 1.48 ^a^	1.35 ± 0.16 ^a,b^	nd	1.18 ± 0.11 ^a^	14.26	4.81
7. RC_SD	685.24 ± 9.28 ^b^	21.20 ± 2.11 ^c,d^	51.27 ± 3.21 ^a,b^	191.78 ± 7.89 ^b^	51.27 ± 7.04 ^b,c^	8.34 ± 1.69 ^b^	1.69 ± 0.41 ^a^	1.11 ± 0.10 ^a^	nd	13.37	4.74

GalA: D-Galacturonic acid; GlcA: D-Glucuronic acid; Rha: L-Rhamnose; Gal: D-Galactose; Ara: L-Arabinose; Glc: D-Glucose; Xyl: D-Xylose; Man: D-Mannose; Fuc: L-Fucose; nd—not determined; the values are presented as mean ± standard deviation; ^a,b,c,d^ Different letters in a column show significantly different values (one-way ANOVA Tukey HSD test: *p* < 0.05).

## Data Availability

Data is contained within the article.
